# Performances of two rapid LAMP-based techniques for the *intrapartum* detection of Group B *Streptococcus* vaginal colonization

**DOI:** 10.1186/s12941-024-00695-2

**Published:** 2024-04-25

**Authors:** Rym Charfi, Cécile Guyonnet, Meiggie Untrau, Gaëlle Giacometti, Thierry Paper, Claire Poyart, Céline Plainvert, Asmaa Tazi

**Affiliations:** 1 Université Paris Cité, CNRS, INSERM, Institut Cochin, Paris, F-75014 France; 2 Service de Bactériologie, Centre National de Référence des Streptocoques, Assistance Publique – Hôpitaux de Paris Centre Université Paris Cité, Hôpital Cochin, 27 rue du Faubourg Saint-Jacques, Paris, 75014 France; 3 Fédération Hospitalo-Universitaire Fighting Prematurity - FHU Préma, Paris, France; 4 Biosynex SA, Illkirch-Graffenstaden, 67400 France

**Keywords:** Group B *Streptococcus*, *Intrapartum* screening, NAAT, Neonatal infection, LAMP

## Abstract

**Purpose:**

Group B *Streptococcus* (GBS) is the leading cause of invasive infections in newborns. The prevention of GBS neonatal disease relies on the administration of an *intrapartum* antibiotic prophylaxis to GBS-colonized women. In recent years, rapid *intrapartum* detection of GBS vaginal colonization using real-time nucleic acid amplification tests (NAATs) emerged as an alternative to antenatal culture screening methods.

**Methods:**

We compared the performances of two loop-mediated isothermal amplification (LAMP) tests, the Ampliflash® GBS and the PlusLife® GBS tests, to standard culture for GBS detection in vaginal specimens from pregnant women. The study was conducted from April to July 2023 in a French hospital of the Paris area.

**Results:**

A total of 303 samples were analyzed, including 85 culture-positive samples (28.1%). The Ampliflash® GBS test and the PlusLife® GBS tests gave a result for 100% and 96.3% tests, respectively. The performances of the tests were as follows: sensitivity 87.1% (95% confidence interval (CI) 78.3–92.6) and 98.7% (95% CI 93.0-99.8), specificity 99.1% (95% CI 96.7–99.8), and 91.9% (95% CI 87.3–95.0), respectively. False negative results of the Ampliflash® GBS test correlated with low-density GBS cultures. Time-to-results correlated with GBS culture density only for the PlusLife® GBS test (*p* < 0.001).

**Conclusion:**

Both techniques provide excellent analytical performances with high sensitivity and specificity together with a short turnaround time and results available in 10 to 35 min. Their potential to further reduce the burden of GBS neonatal disease compared with antenatal culture screening needs to be assessed in future clinical studies.

## Introduction

*Streptococcus agalactiae* also known as Group B *Streptococcus* (GBS) is the worldwide leading cause of invasive infections including sepsis and meningitis in newborns and a major health issue for pregnant and post-partum women [1, 2]. This commensal bacterium colonizes the genitourinary tract of 10–30% women and is responsible for two neonatal syndromes, the early-onset disease (EOD, 0–6 days of life) and the late-onset disease (LOD, 7–89 days old). Importantly, EOD mainly results from maternofetal transmission during parturition and can be prevented by the administration of an *intrapartum* antibiotic prophylaxis (IAP) to GBS-colonized mothers. Therefore, most industrialized countries including the USA and France have implemented a strategy based on the antenatal screening of GBS vaginal or recto-vaginal colonization to identify women candidates for IAP [3]. Overall, strategies based on universal screening of pregnant women resulted in a significant decline in the incidence of EOD in the USA from 1.8 cases/1,000 live births in the early 1990s to 0.26 cases/1,000 live births in 2010, as well as in France [4, 5].

Current American and European guidelines still recommend bacterial culture as the gold standard technique for antenatal GBS screening. The screening has to be performed on vaginal or rectal-vaginal samples between 36 and 37 (American guidelines) or 35 and 37 (European guidelines) weeks of gestational age [6–10]. However, several reports have pointed limitations of this strategy. Because GBS colonization of the genitourinary tract is intermittent, GBS antenatal screening is characterized by a low positive predictive value (PPV) for *intrapartum* GBS colonization, leading to inappropriate IAP in 30–40% cases [11–13]. Similarly, up to 10% of women with a negative antenatal screening turn out positive at delivery and do not benefit from IAP [14]. Last, culture techniques require a minimum of 18 h before giving the first results and are unhelpful in the case of preterm labor and in women without prenatal care. Hence, 60 to 80% EOD cases are born to mothers with a negative antenatal screening or to non-screened mothers [15, 16].

In this context, rapid *intrapartum* detection of GBS colonization emerged as an alternative to antenatal culture screening methods. Although not considered by health authorities as the method of first choice, *intrapartum* GBS testing using rapid and accurate molecular tests has been recommended as the first line screening method by a European consensus conference held in 2013 [17]. These tests are also recommended in women without prenatal care in the USA and Canada. Historically, molecular GBS testing was mainly represented by real-time PCR tests whose analytical and diagnostic performances were assessed in several studies, both as laboratory or point-of-care tests [12, 13, 18–21]. More recently, non-PCR nucleic acid amplification tests (NAATs) such as loop-mediated isothermal amplification (LAMP) assays were developed. These tests which allow a faster detection of pathogens and do not require high-cost laboratory instruments are increasingly used [22]. In this study, we aimed to evaluate the performances of two LAMP-based tests approved by the European Community (CE-IVD, European CE marking for in vitro diagnostic medical devices) for the detection of GBS in vaginal samples, namely the Ampliflash® GBS and PlusLife® GBS tests (Biosynex SA, Illkirch-Graffenstaden, France). We determined the analytical performances of each test compared to that of conventional GBS culture and compared key metrics such as the time-to-result (TTR) related to workflow.

## Materials and methods

### Study design

This single-center retrospective study was conducted in the University Hospital Cochin-Port-Royal between February and April 2023. The maternity ward of the Cochin Hospital is a level III maternity with approx. 5,500 deliveries per year. Non redundant vaginal samples for routine clinical care including GBS antenatal screening between 35 and 37 weeks of gestational age or *intrapartum* samples regardless of gestational age were collected from pregnant women and sent to the laboratory in Amies transport medium (Copan Diagnostics, Brescia, Italy). Samples were immediately processed in the laboratory for GBS detection by culture methods and stored at -80 °C before molecular testing. Based on an expected sensitivity and specificity of 90%, we estimated that a total of 300 samples, including 100 positive samples would be necessary to achieve statistical robustness. Therefore, all positive samples were prospectively included. A selection of negative samples was made each day to achieve a ratio of two negative samples to one positive sample.

### Sample processing

Vaginal swabs were cultured on Columbia agar plates with 5% horse blood incubated under aerobic atmosphere with 5% CO_2_ and on Granada plates incubated under anaerobic atmosphere (bioMérieux, Marcy L’Étoile, France). All plates were examined after 18–24 h incubation at 37 °C, and incubated for an additional 24 h when negative for GBS. β-hemolytic orange pigmented colonies and all suspect colonies including white colonies on Granada plates were identified as GBS by matrix-assisted laser desorption/ionization time-of flight (MALDI-TOF) mass spectrometry (Bruker Daltonics, Billerica, Massachusetts, USA).

To avoid biases due to sample storage at -80 °C, the bacterial culture was repeated in parallel to NAATs testing. Samples were processed in July 2023 immediately after thawing as described above. Besides, 50 µL of samples were inoculated in 9 mL of brain hearth infusion broth for a non-selective enrichment and incubated for 24 h at 37 °C. When samples were negative for GBS after direct plating, a subculture of the enrichment broth was performed on Columbia agar plates with 5% horse blood and on Granada plates as described above. When samples were positive for GBS after direct plating, a semi-quantitative evaluation was performed as follows: GBS colonies on a single quadrant (1), on two quadrants (2), on three quadrants (3), and on four quadrants (4). Samples showing discrepant results regarding the presence or absence of GBS before and after storage at -80 °C were excluded from subsequent analyses.

### Assay procedures

The Ampliflash® GBS and Pluslife® GBS tests were carried out in parallel to GBS culture confirmation on thawed samples in July 2023. Testing was performed by a trained operator according to the manufacturers’ instructions with slight modifications.

Briefly, 50 µL of samples in Amies transport medium were aliquoted for the Ampliflash® GBS test and 250µL for the Pluslife® GBS tests. After centrifugation (14 000 rpm, 5 min), the supernatant was removed and the pellets were resuspended either in 250 µL of the BIOSYNEX IntimaSwab transport medium for the Ampliflash® GBS test or in 250 µL of the PlusLife® GBS test lysis buffer. The following steps were performed according to the manufacturers’ instructions. For the Ampliflash® GBS test, lysis was performed at 98 °C (5 min). Next, 35 µL of lysed samples were mixed with the rehydration buffer of the lyophilized Ampliflash® GBS test and transferred in dedicated wells of the reaction strips. Amplifications were run in Ampliflash® readers, where up to two strips can be placed. For the PlusLife® GBS assay, thermal lysis was performed in the PlusLife Dry Bath incubator at 65 °C (5 min). Samples were transferred to the reaction cards for amplification in the PlusLife instrument where up to eight cards can be placed.

Both tests target the *atr* and *cfb* genes of GBS, which encode the glutamine transporter protein and the CAMP-factor, respectively. The test is interpreted as positive when either of the two targets is amplified, negative when none of the target is amplified, and invalid when the internal control for DNA amplification (ubiquitous human gene encoding the ribonuclease P) is negative. Invalid results may be caused by insufficient DNA concentration due to incorrect sampling or excess inhibitors such as mucus or blood in the vaginal sample. In both systems, amplification results are available in real-time e.g., in 5–30 min and 7–35 min for the Ampliflash® and PlusLife® GBS tests, respectively.

### Data interpretation and control of discrepancies

We used conventional culture as the primary reference method. Discrepant results were controlled by culture and by two additional molecular assays following nucleic acids extraction using the NucleoMag Dx Pathogen kit (MACHEREY-NAGEL, Hoerdt, France). Molecular assays used as controls were the VIASURE *Streptococcus* B Real Time PCR Detection Kit (CERTEST BIOTEC, Zaragoza, Spain), which also targets a conserved region of the *cfb* gene, and a homemade Real Time PCR targeting the GBS *dltR* gene [23].

Because prior studies have suggested that NAATs can be more sensitive than conventional cultures, we also compared the results of each assay to consensus results of each of the four molecular assays. In this case, culture-negative samples were categorized as true positive (TP) if positive by two or more molecular assays [21, 24].

### Statistical analysis

Analytical performances including sensitivity, specificity, PPV and negative predictive value (NPV) were determined by comparison with the reference method e.g., culture with enrichment, and with the consensus results where at least 2/4 NAATs agree. Invalid results and errors were excluded from the statistical analysis. The 95% confidence intervals (CI) for binomial proportions were calculated using the Wilson score method. A McNemar’s chi-square test was performed to compare the performances of the tests. For samples giving positive NAATs results, TTR was correlated to GBS bacterial load by culture using the Kruskal-Wallis test. A *p* value < 0.05 was considered significant.

## Results

### Comparison of the molecular assays to GBS culture

A total of 317 non-redundant vaginal samples were included between February and April 2023. All samples were from pregnant women and were performed as part of routine clinical care and included the detection of GBS by culture methods. A total of 14 samples initially found positive (*n* = 9) and negative (*n* = 5) for GBS by routine bacterial culture showed discrepant results after storage at -80 °C and were excluded from the study. Eventually, a total of 303 samples were analyzed, including 85 culture-positive samples (28.1%). Among these, all but one were positive after direct plating, the latter being positive only after broth enrichment.

Due to insufficient volume, 13 samples could only be tested by a single molecular technique e.g., the Ampliflash® GBS test. Besides, 4 samples could not be tested by the Pluslife® GBS test because of technical problems. Overall, 303 and 286 samples were analyzed by the Ampliflash® GBS and Pluslife® GBS Nucleic Acid test, respectively. The Ampliflash® GBS assay gave a result for all tested samples, showing a rate of invalid tests < 0.4%. This rate was of 3.8% with the PlusLife® GBS test which gave a result for 275 of the 286 samples tested. The results of both NAATs compared to GBS culture are summarized in Table 1. The percent agreement between NAATs was 90.3% (260/288 tests). Of note, the sample which showed a positive GBS culture only after the enrichment step gave a negative and an invalid result with the Ampliflash® and the PlusLife® test, respectively.

**Table 1:** Comparison of NAATs to culture results

Assay	Assay result	Culture result		Enrichment culture result		Total
		Positive	Negative	Positive	Negative	
Ampliflash® GBS	Positive	74	2	74	2	76
	Negative	10	217	11	216	227
PlusLife® GBS	Positive	76	16	76	16	92
	Negative	1	182	1	182	183
	*Invalid*	*2*	*9*	*2*	*9*	*11*
	*Not tested*	*6*	*11*	*6*	*11*	*17*
All assays		84	219	85	218	303

GBS: Group B *Streptococcus*; NAATs: nucleic acid amplification tests

### Analytical performances compared with GBS culture and with consensus results

The analytical performances of both NAATs were first compared to GBS culture as the gold standard reference method (Table 2). The Ampliflash® test showed a sensitivity and a specificity of 87.1% (95% CI 78.3–92.6) and 99.1% (95% CI 96.7–99.8), respectively, compared with 98.7% (95% CI 93.0-99.8) and 91.9% (95% CI 87.3–95.0) for the PlusLife® test, respectively.

**Table 2:** Performances of NAATs compared to culture and consensus results

Assay	Sensitivity (95% CI)	Specificity (95% CI)	Predictive positive value (95% CI)	Predictive negative value (95% CI)	
**Compared to culture results**					
Ampliflash® GBS	87.1% (78.3–92.6)	99.1% (96.7–99.8)	97.4% (90.9–99.3)	95.2% (91.5–97.3)	
PlusLife® GBS	98.7% (93.0-99.8)	91.9% (87.3–95.0)	82.6% (73.6–89.0)	99.5% (97.0-99.9)	
**Compared to consensus results**					
Ampliflash® GBS	87.4% (78.8–92.8)	100% (98.3–100)	100% (95.2–100)	95.2% (91.5–97.3)	
PlusLife® GBS	98.7% (93.2–99.8)	92.9% (88.4–95.7)	84.8% (76.1–90.7)	99.5% (97.0-99.9)	
Culture	97.7% (92.0-99.4)	100% (98.3–100)	100% (95.7–100)	99.1% (96.7–99.8)	

CI: confidence interval; GBS: Group B *Streptococcus*; NAATs: nucleic acid amplification tests

Next, we sought to investigate the false positive results using two additional PCR tests. The two false positives of the Ampliflash® test were also detected positive by the three other NAATs e.g., the PlusLife® assay and the two control PCR tests. Accordingly, the samples were classified as positive by the consensus method. A total of 14 more samples were classified as false positives with the PlusLife® test using culture as the reference method. The control PCR tests gave a negative result for all of these samples, with the exception of one which was therefore classified as positive by the consensus method.

Overall, the PlusLife® GBS test was significantly more sensitive than the Ampliflash® GBS test compared with culture and with consensus results (McNemar’s chi-square test, *p* = 0.021). Conversely, the Ampliflash GBS test was significantly more specific than the PlusLife® GBS test (McNemar’s chi-square test, *p* < 0.001). Compared to consensus results, the PPV and NPV of the Ampliflash® test were of 100% (95% CI 95.2–100) and 95.2% (95% CI 91.5–97.3), respectively, whereas those of the PlusLife® test were 84.8% (95% CI 76.1–90.7) and 99.5% (95% CI 97.0-99.9), respectively.

### Concordance between NAATs results and GBS culture density

To better characterize NAATs performances, we analyzed false negative results with respect to GBS load as assessed by semi-quantitative bacterial culture. Taking the consensus results as a reference, among the 12 Ampliflash® GBS false negative results, 1 was negative by culture, 1 was positive only after enrichment, 8 were associated with a low GBS load (1 quadrant), and 2 with a moderate GBS load (2 quadrants). Notably, only 5 out of these 12 samples were detected positive with the commercial PCR test used as a control. The threshold cycles (Ct) observed with this latter method were > 35, indicative of a low DNA load. The PlusLife® GBS test gave only one false negative result which was associated with a high GBS load by culture (4 quadrants). All other NAATs detected this sample as positive for GBS. Unfortunately, the PlusLife® GBS test could not be repeated and a technical error cannot be excluded in this particular case.

**Fig. 1:**
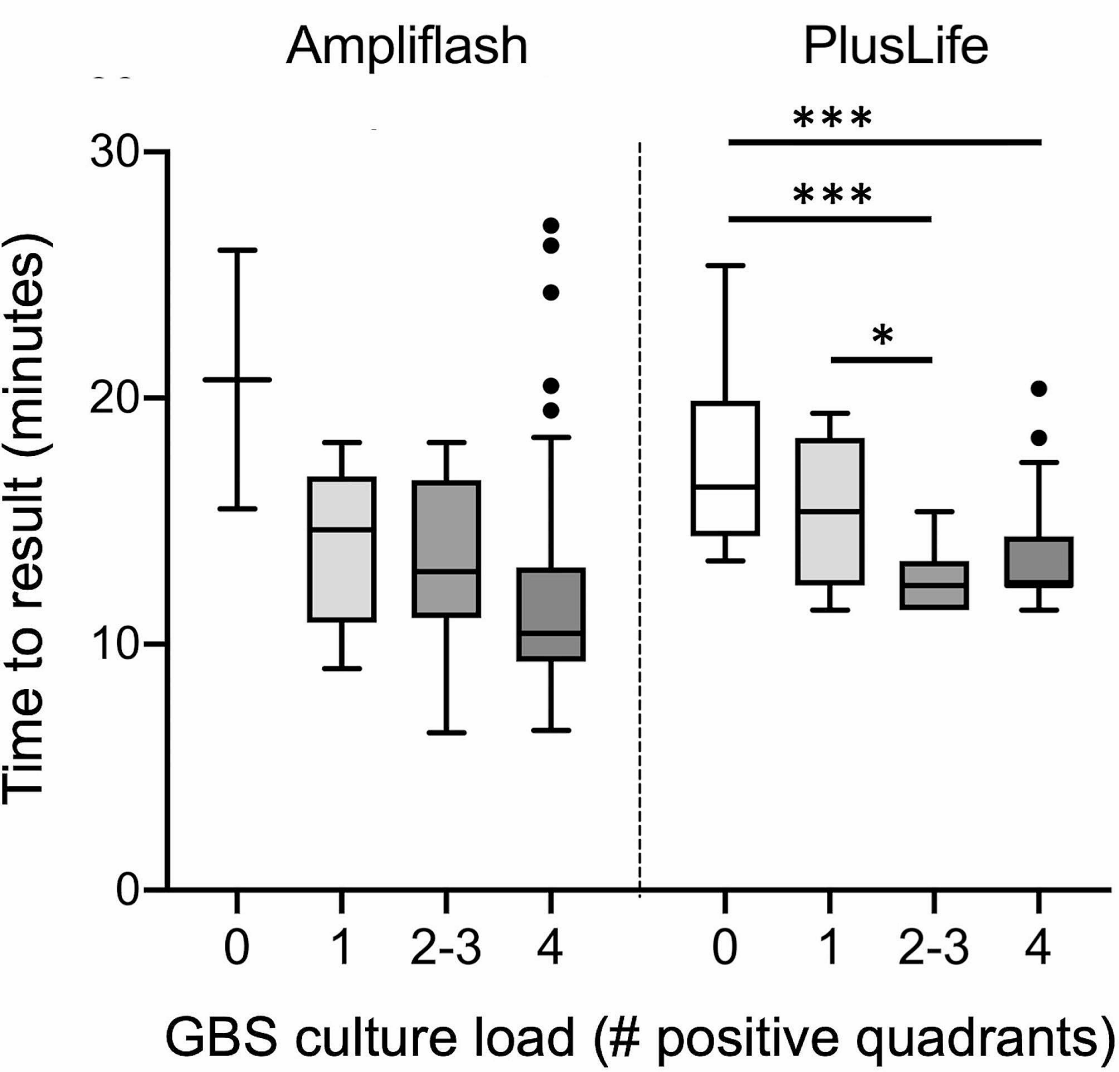
Time-to-result in minutes for GBS detection according to GBS density in culture The bottom, middle, and top lines of each box plot correspond to the 25%, 50%, and 75% cumulative frequencies of the observed values, respectively. The endpoints of the whiskers show the 2.5th and 97.5th percentiles. GBS culture load was categorized as follows: (0) no GBS, (1) colonies on a single quadrant, (2–3) colonies on two to three quadrants, and (4) colonies on four quadrants. The number of specimens for the Ampliflash® test and the PlusLife® test by GBS culture category was (0) 2 and 16, (1) 8 and 13, (2–3) 16 and 16, and (4) 52 and 47, respectively. Statistical analysis was performed using the Kruskall-Wallis test. * *p* < 0.05, *** *p* < 0.0001

Next, we analyzed the correlation between TTR and the GBS load observed in culture. The median TTR was 12 (range 6–27) and 13 (range 11–25) min for the Ampliflash® and Pluslife® GBS tests, respectively. The TTR was not significantly correlated with GBS culture density for the Ampliflash® test (Fig. 1). Conversely, for the Pluslife® test, the TTR corresponding to GBS-negative cultures (*n* = 16, including 13 false negatives and 3 true positives according to consensus results) and to GBS low-density cultures was higher than that of GBS mild- to highly-positive cultures.

## Discussion

In the early 2000s, the widespread implementation of preventive strategies based on GBS antenatal screening and IAP for colonized women resulted in a substantial decline of GBS EOD in several industrialized countries [4]. Whereas universal culture-based screening in late pregnancy between 35 or 36 and 37 weeks of gestational age remains the gold standard in the USA, Canada, and Europe, the European consensus conference recommends *intrapartum* screening using NAATs for all women, except in case of beta-lactam allergy where GBS isolation and antibiotic susceptibility testing are required [6–10, 17]. Although previous studies reported lower sensitivity of NAATs compared with culture for GBS detection, *intrapartum* NAATs showed better sensitivity and PPV than antenatal culture screening for the detection of *intrapartum* GBS colonization [19, 25, 26]. Besides, in more recent studies, several NAATs proved more sensitive than the gold standard enrichment culture but only when both NAAT and culture were performed after an enrichment step [21, 24, 27].

In the present study, we compared the performances of two European Community approved NAATs assays with culture for GBS detection in vaginal specimens from pregnant women. Our main finding was that both tests displayed very good to excellent analytical performances and that they both met the criteria specified by the European consensus conference for such tests e.g., a short turnaround time < 45 min, and accuracy with high sensitivity and specificity, not inferior to 90–95% and 95–98% respectively [17]. Nevertheless, whereas the Ampliflash® test had a very high specificity, its sensitivity was at the threshold limit (87.4%, 95% CI 78.8–92.8), and *vice-versa* for the PlusLife® test which also displayed a specificity at the threshold limit (92.9%, 95% CI 88.4–95.7). These performances are likely linked to the technology used in each of the two tests and to the technical procedure. The Ampliflash® GBS test is a typical LAMP assay and was expected to be highly sensitive. However, its sensitivity was lower than that of culture, while remaining similar to that of the PCR tests used as controls. This finding might be related to the adaptation of the technical procedure that had to be carried out, in which the vaginal specimens were not discharged in the dedicated transport medium supplied with the test. Of note, false negative results were mainly associated with low bacterial densities in culture or PCR, which are less at risk of contaminating neonates and also, considering bacterial densities in culture, less at risk of maternal and neonatal infection [28–30].

The PlusLife® GBS assay is an RNase Hybridization-Assisted amplification (RHAM) assay, which is a novel technology where LAMP is combined with an RNase HII-mediated fluorescent reporter system presumed to increase both sensitivity and specificity [31]. However, taking either culture or the consensus results as the reference methods, the PlusLife® GBS assay had the lowest specificity of all tests. The potential false positives correlated to high TTR values, which as high Ct values are often associated with false-positive results [27]. These results, however, could also represent low-level positives that could not be detected by the other NAATs or by culture. In the absence of an alternative highly sensitive technique, and of experience with this novel technology, it appears difficult to draw any categorical conclusions.

In contrast to other studies, taking the consensus results as the reference method, NAATs did not significantly improve GBS detection over culture [19, 31]. For instance, the consensus method added only three positive specimens to the 85 previously identified by culture. All three specimens showed high Ct values between 33.6 and 37.2 with the commercial PCR test used as a control, indicative of a low bacterial load. Other studies demonstrated that NAATs had significantly higher sensitivity than culture by up to 40%, but only when performed after a step of enrichment in a selective broth [21, 27]. Hence, our findings which show lower sensitivity of NAATs compared to culture are in agreement with those reported elsewhere [19, 25, 32]. In addition, the sensitivity of the Ampliflash® GBS test in our study is similar to that reported in a recent study conducted in the Democratic Republic of Congo which found a sensitivity of 96.6% and 87.5% compared with a reference qPCR performed on vaginal samples with Ct ≤ 33 and < 40, respectively [33]. In addition to technical issues that we discussed above, reduced sensitivity of NAATs compared with culture may be due to technical sampling issues in the particular circumstances of *intrapartum* testing such as ruptured membranes or vaginal bleeding which could decrease bacterial density and lead to false negative results. Besides, while most studies use blood agar plates for the detection of GBS, we used Granada agar plates, which enhances GBS detection in polymicrobial specimens such as vaginal specimens [34, 35]. The major limitation when using blood agar plates is that approximately 5–8% of GBS are not hemolytic and may be unrecognized [36, 37]. Isolated on Granada medium, beta-hemolytic GBS appear as pigmented orange to red colonies, whereas non beta-hemolytic isolates grow as white colonies. In addition to the fact that beta-hemolytic GBS are easily distinguished on Granada medium, the latter is selective for streptococci and enterococci. Thus, testing of all white colonies enable all GBS isolates to be recognized, likely enhancing culture sensitivity.

According to the European consensus conference, in addition of being rapid and accurate, *intrapartum* GBS tests should also be easy to perform and to interpret, and available at all times 24 h a day, seven days a week [17]. The Ampliflash® GBS test and the PlusLife® GBS test display distinct features that can be used in different ways depending on the needs and on the clinical and laboratory settings. The Ampliflash® GBS test requires approx. 10 min of technical handling including pipetting before the amplification step is started. This latter step can be performed either in the Ampliflash® reader or in any open real-time thermal cycler. Conversely, the PlusLife® test can be performed without any specific equipment or environment. While the former test is primarily intended for laboratory use, the latter could easily be used as a point-of-care test, in delivery rooms. Although the PlusLife® GBS test had a higher rate of invalid results, this rate remained lower than 4% which seems acceptable and similar to that previously reported for other NAATs [12, 13, 21].

Our study provides a first evaluation of the analytical performances of two innovative molecular techniques for *intrapartum* GBS screening. Nevertheless, certain limitations should be highlighted. First of all, this was a retrospective study and the vaginal specimens were stored at -80 °C before being tested, which might have induced bacterial and nucleic acid degradation, and biases in the sensitivities and specificities we report. Secondly, this was a single-center study where all the tests were carried out by trained and experimented staff, which likely improved the sensitivity of GBS detection by culture and NAATs. Last, as all tests were performed on a single swab, some discrepancies could not be investigated due to insufficient volume.

## Conclusion

Since the implementation of universal GBS antenatal screening and IAP for GBS-colonized mothers in the early 2000s, the incidence of GBS EOD rapidly deceased before reaching a plateau in the mid-2010s, pointing at the limitations of this strategy and shedding the light on the need for alternatives. On the one hand, a GBS vaccine for pregnant women would offer high potential for reducing the burden of GBS maternal and neonatal infections, but it is not yet available [38, 39]. On the other hand, highly sensitive *intrapartum* NAATs including LAMP-based assays could also further reduce the burden of GBS neonatal disease by increasing the sensitivity for its detection compared with antenatal culture screening and decreasing turnaround times. This study demonstrates that the Biosynex Ampliflash® GBS and the PlusLife® GBS tests possess excellent analytical performances and a fast, simple workflow for rapid GBS screening in vaginal samples. Further prospective studies to address the feasibility and clinical impact of *intrapartum* NAATs compared to conventional culture methods are needed to assess their potential benefits in preventing GBS EOD, neonatal mortality and morbidity, and more generally, to evaluate their medico-economic impact.

## Data Availability

The data generated during the current study are available from the corresponding author on request.
